# Perks of being a wallflower: a high-quality wallflower reference genome reveals its chromosome evolution and flower color variation

**DOI:** 10.1093/plphys/kiag150

**Published:** 2026-03-18

**Authors:** William J W Thomas

**Affiliations:** Assisstant Features Editor, Plant Physiology, American Society of Plant Biologists; School of Biological Sciences, The University of Western Australia, Perth, Western Australia, Australia

Wallflowers are so named because of their tendency to grow in rocky crevices or up walls. Part of the diverse mustard family (Brassicaceae), wallflowers are native to southeast Europe. They are popular garden plants but are also commonly found in the wild. Wallflowers grow in a range of vivid colors, making them bright and vibrant, unlike their shy and introverted human namesake (Fig. 1). Not surprisingly, they are important ornamental plants, with different cultivars displaying dramatic color combinations. This is exemplified by aptly named cultivars such as “Fire King,” “Sunset Apricot,” and “Night Skies.” Most cultivars are the species *Erysimum cheiri*, which is 1 of more than 270 within the *Erysimum* genus. Genetic studies have revealed that these species vary in chromosome numbers, ranging from 6 to 17 chromosomes, reflecting complex evolutionary histories. *E. cheiri* also contains medicinal metabolites with anti-inflammatory and anti-tumor properties, attracting recent interest in the species for potential disease treatment (Mosleh et al. 2021). However, to date there is only one *E. cheiri* reference genome, which was constructed with short-reads and is not assembled at the chromosome level. The lack of a high-quality reference genome for *E. cheiri* has hampered understanding of its evolution and the genetic basis of important traits.

**Figure 1:**
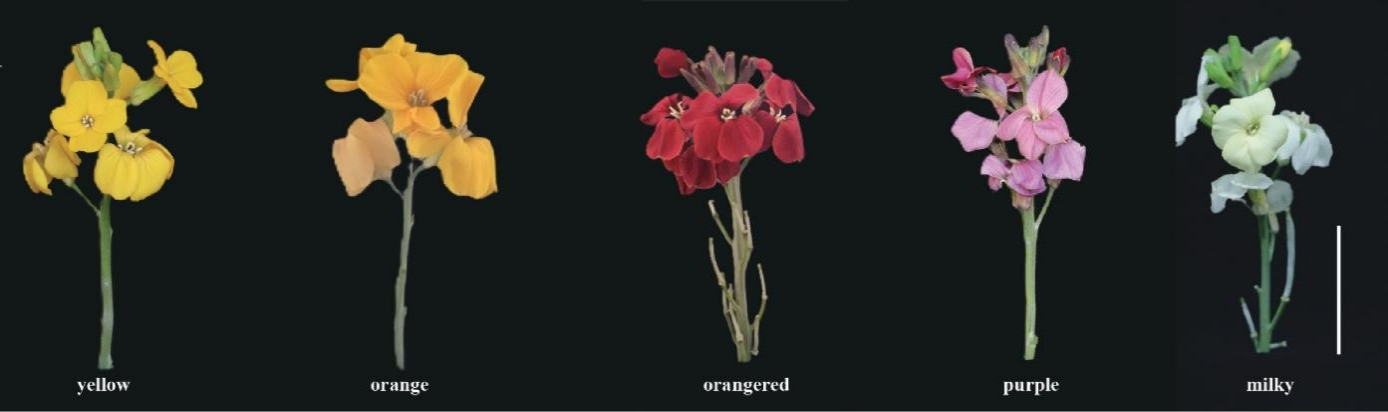
Five differently colored *E. cheiri* plants utilized to study flower color variation by Chen et al. (2026). Scale bar is 3 cm. Figure from Chen et al. (2026).

Recently in *Plant Physiology*, Chen et al. (2026) generated a near telomere-to-telomere reference genome of *E. cheiri*. They used this to investigate the evolution of chromosomes and the involvement of specialized metabolites in determining flower color. The genome was constructed using different technologies, including PacBio HiFi long-read and Illumina short-read sequencing, and Hi-C scaffolding. These were combined to generate a 244-Mb genome sorted into 6 chromosomes. The contiguity of the assembly was relatively high (N50 = 44 Mb)—a 500-fold improvement over the previous *E. cheiri* reference genome (Kiefer et al. 2019). The authors identified 38,788 protein-coding genes by integrating different gene prediction methods. BUSCO analysis, which measures genome completeness, indicated that their newly assembled genome captured 99.5% of the expected core genes that are conserved among related species. Together, these metrics reflect a markedly improved high-quality reference genome for the wallflower.

With this new genome, Chen et al. (2026) were able to trace the evolutionary changes in chromosome structure that explain the 6 chromosomes defined in *E. cheiri*. This was done by comparative analysis using the genome of a closely related species, *E. cheiranthoides*, which has 8 chromosomes instead of 6 (Zhai et al. 2024). The authors reconstructed the origin of *E. cheiri*'s 6 chromosomes and found that 2 (E01 and E02) were much longer compared to their *E. cheiranthoides* counterparts. On further investigation, E01 and E02 were identified as fusion chromosomes, where 2 ancestral chromosomes fuse to form a single chromosome, much like tying 2 short pieces of string together to make a longer one. This is a classic example of descending dysploidy, when chromosome numbers are reduced over evolutionary time by chromosomal rearrangements (Mandáková and Lysak 2018). Usually, genomic information is not lost during this process but shuffled around and structured in different ways. These findings unveil a new understanding of the mechanisms behind *Erysimum* genome evolution and provide a framework for exploring chromosome evolution more broadly across the genus.

Another objective of the study was to determine how specialized metabolites influence flower color variation in the wallflower. Specialized metabolites, including flavonoids and carotenoids, are known to be responsible for petal pigmentation in flowering plants (Rezende et al. 2025). However, the role of these metabolites in *E. cheiri* has not been extensively characterized. Chen at al. (2026) analyzed the metabolite profile of five *E. cheiri* plants with distinct flower colors (Fig. 1). They identified 78 flavonoid and 68 carotenoid metabolites across the samples. The number of anthocyanin metabolites, a subclass of flavonoids, varied between the different colors, whereas the number of carotenoids was relatively similar. By assessing differential metabolites between samples, the authors found that the accumulation of carotenoids is mainly responsible for yellow and milky colors, while the accumulation of flavonoids produces purple, orange, and orange-red colors. The fact that both carotenoid and flavonoid biosynthetic pathways are active in *E. cheiri* means that interacting metabolites produce a co-coloring effect. This explains the diverse range of colors exhibited by the wallflower.

Finally, Chen et al. (2026) conducted RNA-sequencing of the differently colored petals to understand the genetic mechanisms underpinning flower color variation. Transcriptomic analysis revealed 297 differentially expressed genes (DEGs) shared between different color groups. These DEGs were enriched in the biosynthetic pathways of anthocyanin and other specialized metabolites, further supporting their involvement in petal pigmentation. In-depth network modeling enabled the authors to link hub genes within the flavonoid and carotenoid biosynthetic pathways with metabolite profiles. They identified 12 genes strongly associated with flavonoids and 5 strongly associated with carotenoids, all of which were significantly differentially expressed among the different colors. These results shed light on the genetic basis of flower color variation through changes in expression levels of genes involved in specialized metabolite biosynthesis.

The work by Chen et al. (2026) presents, to our knowledge, the first chromosome-level reference genome for the important ornamental species *E. cheiri* and an understanding of its chromosome evolution and molecular insights into flower color variation. The near-gapless reference genome is a foundational resource in understanding the evolutionary history of *Erysimum* species and for the molecular breeding and improvement of the wallflower. For example, it has promising applications in the genetic manipulation of specialized metabolite biosynthesis to produce new and exotic flower colors—a highly sought-after and economically valuable trait. To this end, the genes identified in this research are potential targets for further investigation. The availability of a high-quality wallflower genome also sets the stage for more detailed research into the medicinal applications of certain metabolites found in *E. cheiri*.

## Data Availability

No new data were generated or analyzed in support of this article.

## References

[kiag150-B1] Chen D et al 2026. Insights into karyotype evolution and flower color variation from the genome assembly of wallflower (*Erysimum cheiri*). Plant Physiol. 10.1093/plphys/kiag133. PMC13089509 41824659

[kiag150-B2] Kiefer C et al 2019. Interspecies association mapping links reduced CG to TG substitution rates to the loss of gene-body methylation. Nat Plants. 5:846–855. 10.1038/s41477-019-0486-9. 31358959

[kiag150-B3] Mandáková T, Lysak MA. 2018. Post-polyploid diploidization and diversification through dysploid changes. Curr Opin Plant Biol. 42:55–65. 10.1016/j.pbi.2018.03.001. 29567623

[kiag150-B4] Mosleh G, Azadi A, Khademian S, Heidari R, Mohagheghzadeh A. 2021. Anti-inflammatory activity and quality control of *Erysimum cheiri* (L.) Crantz. Biomed Res Int. 2021:5526644. 10.1155/2021/5526644. 34212031 PMC8208854

[kiag150-B5] Rezende FM, Rossi M, Furlan CM. 2025. Molecular control of flower colour change in angiosperms. Plants. 14:2185. 10.3390/plants14142185. 40733424 PMC12299958

[kiag150-B6] Zhai D et al 2024. Reciprocal conversion between annual and polycarpic perennial flowering behavior in the Brassicaceae. Cell. 187:3319–3337. 10.1016/j.cell.2024.04.047. 38810645

